# PRDM1 rs2185379, unlike BRCA1, is not a prognostic marker in patients with advanced ovarian cancer

**DOI:** 10.3233/CBM-230358

**Published:** 2024-07-01

**Authors:** Klara Horackova, Michal Vocka, Sarka Lopatova, Petra Zemankova, Zdenek Kleibl, Jana Soukupova

**Affiliations:** a First Faculty of Medicine, Institute of Medical Biochemistry and Laboratory Diagnostics, Charles University and General University Hospital in Prague, Prague, Czech Republic; b Department of Oncology, First Faculty of Medicine, Charles University and General University Hospital in Prague, Prague, Czech Republic; c Institute of Pathological Physiology, First Faculty of Medicine and General University Hospital in Prague, Prague, Czech Republic

**Keywords:** Ovarian cancer, long-term survival, PRDM1, BRCA1, biomarker

## Abstract

**BACKGROUND:**

Ovarian cancer (OC) is mostly diagnosed in advanced stages with high incidence-to-mortality rate. Nevertheless, some patients achieve long-term disease-free survival. However, the prognostic markers have not been well established.

**OBJECTIVE:**

The primary objective of this study was to analyse the association of the suggested prognostic marker rs2185379 in *PRDM1* with long-term survival in a large independent cohort of advanced OC patients.

**METHODS:**

We genotyped 545 well-characterized advanced OC patients. All patients were tested for OC predisposition. The effect of *PRDM1* rs2185379 and other monitored clinicopathological and genetic variables on survival were analysed.

**RESULTS:**

The univariate analysis revealed no significant effect of *PRDM1* rs2185379 on survival whereas significantly worse prognosis was observed in postmenopausal patients (HR = 2.49; 95%CI 1.90–3.26; p= 4.14 × 10^ - 11^) with mortality linearly increasing with age (HR = 1.05 per year; 95%CI 1.04–1.07; p= 2 × 10^ - 6^), in patients diagnosed with non-high-grade serous OC (HR = 0.44; 95%CI 0.32–0.60; p= 1.95 × 10^ - 7^) and in patients carrying a *gBRCA1* pathogenic variant (HR = 0.65; 95%CI 0.48–0.87; p= 4.53 × 10^ - 3^). The multivariate analysis interrogating the effect of *PRDM1* rs2185379 with other significant prognostic factors revealed marginal association of *PRDM1* rs2185379 with worse survival in postmenopausal women (HR = 1.54; 95%CI 1.01–2.38; p= 0.046).

**CONCLUSIONS:**

Unlike age at diagnosis, OC histology or *gBRCA1* status, rs2185379 in *PRDM1* is unlikely a marker of long-term survival in patients with advance OC.

## 1. Introduction

Ovarian cancer (OC) is mostly diagnosed in advanced stages (70%), leading to a high incidence-to-mortality rate. Although patients with advanced OC achieve remission with maximum debulking surgery and chemotherapy, recurrence, usually incurable, often occurs within 3 years and the 5-year survival of late-stage OC is below 30% [1]. Nevertheless, some OC patients achieve long-term disease-free survival despite the diagnosis at advanced stage. Thus, attempts are being made to identify further prognostic markers of long-term survival. Recently, the polymorphism rs2185379 in *PRDM1* has been associated with long-term recurrence-free survival in Japanese advanced OC patients and, based on the mouse model, heterozygous rs2185379 was suggested to induce initial differentiation of T lymphocytes in antitumor immune response [2]. However, the precise impact of rs2185379 on protein function or possible linkage to another variant with prognostic significance is unknown. The minor allele frequency of rs2185379 in GnomAD varies between populations from 2% in Latino America to 3.1% in European non-Finnish, 5.9% in East Asian and 7% in African American.

The *PRDM1* (positive regulatory domain zinc finger protein 1; OMIM*603423) codes for the BLIMP1 (B lymphocyte-induced maturation protein 1) transcription factor that is involved in the regulation of antitumor immunity. It was recently shown that BLIMP1 enhances transcription of USP22 deubiquitinating enzyme leading to decreased degradation of SPI1 transcription factor and subsequent enhanced expression of programmed death ligand 1 (PD-L1), which leads to infiltrated CD8+ T cell exhaustion and memory responses [3]. BLIMP1 was shown to play a role in the development of malignant lymphoma, leukemia, and some non-haematopoietic cancers, including breast and colorectal cancer, hepatocellular carcinoma, or glioma as reviewed in [4]. In particular, decreased expression of *PRDM1* correlates with a poor prognosis in lung cancer [5]. However, little is known about the role of *PRDM1* in ovarian cancer. Zhang et al. suggested that tumour-infiltrating T lymphocytes can improve the long-term outcome of patients with advanced OC [6].

In this work, we explored the association of heterozygous rs2185379 *PRDM1* variant with long-term survival of advanced OC and compared the effect of rs2185379 with selected clinicopathological and genetic factors influencing the prognosis of OC patients.

**Table 1:** Clinicopathological characteristics and identified genotypes

		N= 555	N= 182	N= 237	N= 508	N= 37
		All OC pts	Survival < 5y^∗^	Survival > 5y^∗^	rs2185379 -	rs2185379 +
	Mean age at dg	57.5	58.3	54.2	55	55.5
Menoactivity	Pre	165	51	92	149	12
	Post	389	131	145	358	25
	NA	1	0	0	1	0
Histology	Clear cell	3	0	0	3	0
	Endometrioid	15	3	8	13	1
	Mucinous	5	1	3	5	0
	Other	25	8	14	22	2
	Serous	497	165	208	456	34
	- HG	447	154	178	408	33
	- LG	45	9	25	43	1
	- NA	5	2	5	5	0
	Undifferentiated	6	4	2	5	0
	NA/unclassified	4	1	2	4	0
Grade	Poorly differentiated (Grade3, High)	489	170	197	446	34
	Well differentiated (Grade1, Low)	52	10	31	48	3
	NA	14	2	9	14	0
Stage	IIIA	52	10	26	50	1
	IIIB	72	17	36	69	2
	IIIC	349	123	152	313	30
	IV	82	32	23	76	4
Surgery	Primary surgery	343	87	176	316	22
	Interval Debulking	202	89	60	183	15
	No	9	6	1	8	0
	NA	1	0	0	1	0
Residual disease	Not reported	371	86	176	341	26
	Tumour < 1 cm	80	45	22	70	7
	Tumour > 1 cm	70	34	20	65	3
	NA	34	17	19	32	1
Vital status	Alive in complete remission	249	x	145	223	22
	Alive with disease	53	x	26	52	1
	Dead	248	182	66	229	14
	Missing	5	0	0	4	0
Chemo	Adjuvant only	331	85	167	305	21
	Chemo yes, no operation performed	7	6	0	6	0
	NA	7	0	5	7	0
	Neoadjuvant	203	89	61	184	15
	No	7	2	4	6	1
Mutation	neg	369	125	138	342	24
	BRCA1	112	33	63	100	7
	BRCA2	48	17	22	42	5
	RAD51C/D/BRIP1	14	2	9	13	1
	MMR	1	1	0	1	0
	Other (PALB2, ATM, CHEK2, TP53)	11	4	5	10	0
	MINAS	4	3	1	4	0
PRDM1	rs2185379 +	37	9	15		
	rs2185379 -	508	168	219		
	rs2185379_NA	10	5	3		

^*^Alive patients diagnosed in 2018 or later (e.g. < 5y since the OC diagnosis) were excluded.

## 2. Methods

### 2.1 Patients

Five-hundred-and-fifty-five patients diagnosed with advanced staged OC (FIGO stages III/IV) with available DNA were enrolled regardless of familial cancer history or OC histology (Table 1). All the patients were previously tested for OC cancer predisposition [7]. Genotyping of *PRDM1* rs2185379 was successfully performed in 545 of them. Clinicopathological data were obtained during genetic counselling or retrieved from patients’ records. Vital status for the estimation of survival function using the Kaplan-Meier curve was available for 541 patients. All patients were Caucasians of Czech origin. Written informed consent was obtained from all patients. The study was approved by the Ethics Committee of the General University Hospital in Prague (approval number 92/14) and performed in accordance with the Declaration of Helsinki.

### 2.2 Genotyping

We performed genotyping of rs2185379 (NM_001198: c.220G>A; p.Gly74Ser) from DNA derived from peripheral blood using the high resolution melting analyses (primers: 5'-GTGGACAGAGGCTGAGTTTGA-3'; 5’-TCACTGTTGGTGGCATACTTGA-3') on LightCycler 480 System (Roche). Each run included negative and positive control with genotype previously confirmed by whole exome sequencing. All positive samples were confirmed by Sanger sequencing.

### 2.3 Statistical analysis

The effect of monitored variables on survival was analysed using Kaplan-Meier analysis and Cox regression in R studio (libraries survival, ranger, ggplot2, ggfortify). P-values less than 0.05 were considered statistically significant.

## 3. Results

We performed genotyping of 545 well-characterized advanced-stage OC patients that were previously tested for OC cancer predisposition [7]. We identified 37 (6.8%) OC patients heterozygous for rs2185379 and no homozygote of the alternative allele (consistent with Hardy-Weinberg equilibrium). 

**Figure 1.**
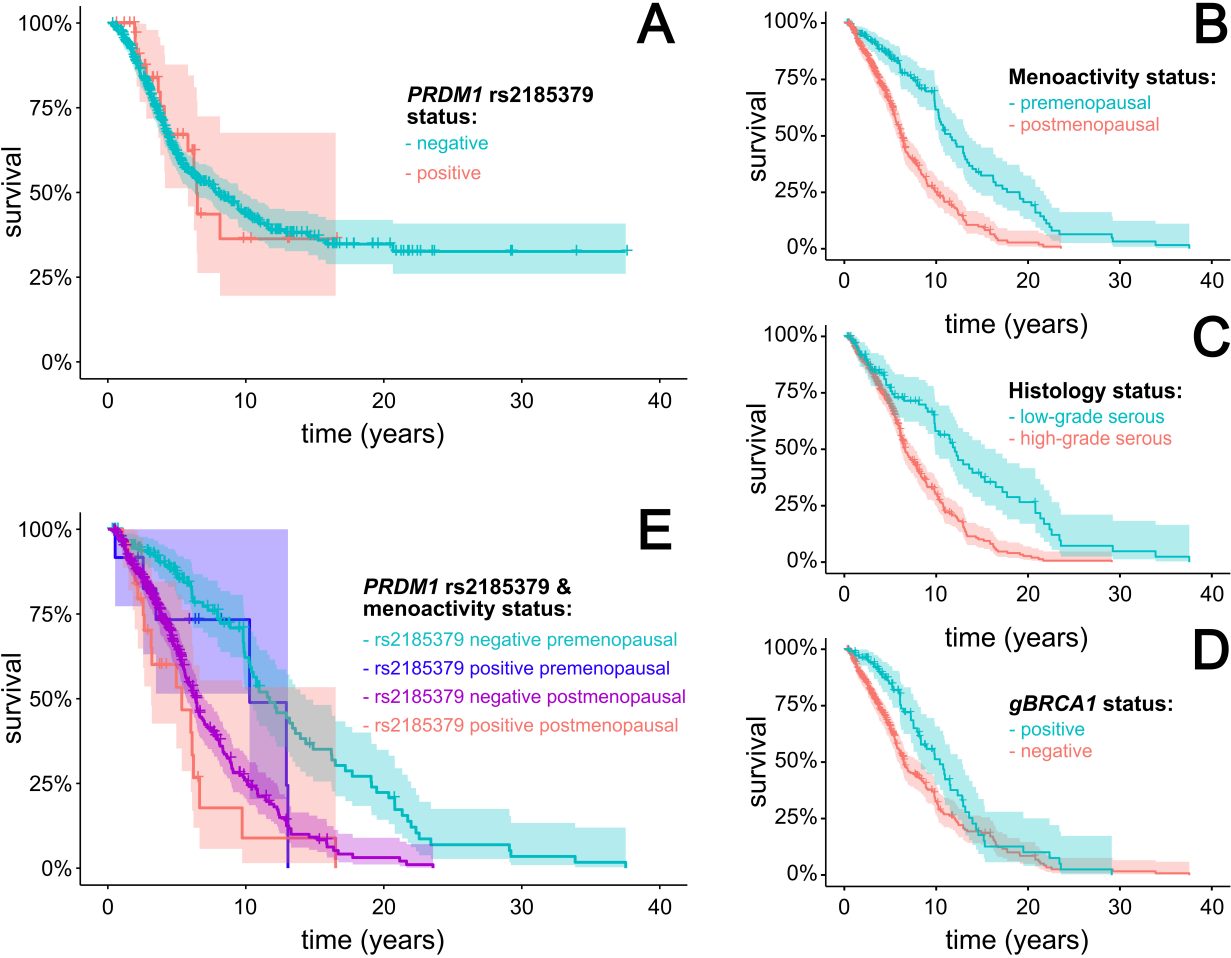
The effect of monitored variables on survival of patients diagnosed with advanced OC. Figure 1 shows univariate survival analysis of the *PRDM1* rs2185379 status (Fig. 1A), menoactivity status (Fig. 1B), OC histology (Fig. 1C), germline *BRCA1* mutation status (Fig. 1D), and multivariate analysis interrogating the effect of *PRDM1* rs2185379 and the menoactivity status (Fig. 1E).

Subsequently, we performed univariate survival analysis of *PRDM1* rs2185379 status as well as of the individual clinicopathological characteristics and presence of pathogenic/likely pathogenic variants in OC predisposition genes. Despite the numerically higher frequency of *PRDM1* rs2185379 heterozygotes among long-term survivors (6.4%) compared to short-term survivors (5.1%; p= 0.3), the heterozygosity of the *PRDM1* rs2185379 was not associated with survival (Fig. 1A). Of the monitored clinicopathological characteristics, survival was significantly associated with menoactivity status at the age at diagnosis with hazard ratio (HR) 2.49 (95%CI 1.90–3.26; p= 4.14 × 10^ - 11^ Fig. 1B) in postmenopausal patients. The risk of mortality increased linearly with increasing age at diagnosis (HR = 1.05 per year; 95%CI 1.04–1.07; p= 2 × 10^ - 6^), without an apparent cut-off point. Furthermore, patients diagnosed with LG serous or overall non-HG serous OC had significantly better survival compared to HG serous OC (HR = 0.48; 95%CI 0.32–0.71; p= 4 × 10^ - 4^; Fig. 1C) and (HR = 0.44; 95%CI 0.32–0.60; p= 1.95 × 10^ - 7^), respectively. In addition, we observed better survival in *BRCA1* mutation carriers compared to non-carriers (HR = 0.65; 95%CI 0.48–0.87; p= 4.53 × 10^ - 3^ Fig. 1D) but this advantage gradually decreased, HRs levelled off around 11 years after diagnosis and then the trend reversed.

The multivariate analysis interrogating the effect of *PRDM1* rs2185379 with other analysed significant prognostic factors revealed that *PRDM1* rs2185379 was marginally associated with worse survival in postmenopausal women with hazard ratio 1.54 (95%CI 1.01–2.38; p= 0.046 Fig. 1E). A similar, but nonsignificant, association of this polymorphism with worse survival was observed in *BRCA1* carriers (HR = 2.3; p= 0.07).

## 4. Discussion

Identification of the genetic and non-genetic factors modulating the prognosis of patients with advanced OC is an important prerequisite for improving the unsatisfactory outcomes of these patients. Here, we analyzed the rs2185379 in the *PRDM1* gene in 545 well-characterized advanced OC patients. Recently, Mitamura and colleagues described association of rs2185379 with an excellent OC prognosis and suggested that the *PRDM1* polymorphism is involved in the anticancer T-lymphocyte immunity [2]. Contrary to this report that analyzed only a small group of 24 advanced OC patients of the Japanese origin, we found lack of the association between rs2185379 and a long-term survival in our 545 Caucasian OC patients of the Czech origin.

To demonstrate the consistency of our patient population, we analyzed previously described associations of monitored clinicopathological and genetic factors with survival in advanced OC. We observed significant survival advantage in patients diagnosed with advanced OC premenopausally, as described by Chan et al. previously [8]. Accordingly, the increasing age at diagnosis directly correlated with worse survival. Similarly, HG serous OC was associated with significantly worse prognosis compared to LG serous or to non-HG serous OC, as described in previous studies [9]. Furthermore, we observed significantly improved survival in *BRCA1*-positive OC patients, with the most pronounced effect in the first five years after diagnosis that disappeared after 11 years since diagnosis. Similar results were observed by McLaughlin et al. and Heemskerk-Gerritsen et al, who observed survival benefit for *BRCA1*- and *BRCA2*-positive OC patients that disappeared after 10 and 6 years after diagnosis, respectively [10, 11].

Interrogation of significant clinicopathological and genetic factors revealed a worsened survival in a subset of rs2185379 carriers diagnosed with advanced OC postmenopausally, suggesting rather an opposite, if any, effect of this genetic marker on advanced OC prognosis. The *PRDM1* gene product BLIMP1 might improve survival and therapeutic response enhancing the transcription of PD-L1 [3]. Blocking PD-1/PD-1L signaling improves anticancer T-cell responses making *PRDM1* a promising survival biomarker. However, rs2185379 in *PRDM1* does not seem to influence the BLIMP1 function significantly, at least with its impact on survival in patients with advanced OC.
